# The Antiseptic Virtues of Vegetable Acid and Marine Salt Combined, in Various Disorders Accompanied with Putridity

**Published:** 1787

**Authors:** William Wright

**Affiliations:** Trelawney, in Jamaica


XIII. *The antiseptic Virtues of vegetable Acid and marine Salt combined, in various Disorders accompanied with Putridity; communicated in a Letter to* John Morgan, *M. D., F. R. S., and Professor of the Theory and Practice of Physic at Philadelphia, by* William Wright, *M. D., of Trelawney, in Jamaica.*—From the *Transactions of the American Philosophical Society,* Vol. II.

HAVING experienced the virtues of vegetable acid and marine salt, when combined, I beg leave to lay before you a few observations on the use of this simple medicine in several diseases. It is my sincere wish, that it may prove as beneficial to mankind in general, as it has been to many of my patients in this part of the country.

Take of lime juice, or lemon juice, three ounces; of marine salt as much as the acid will dissolve; of any simple distilled cordial water, one pint; and of loaf sugar a sufficient quantity to sweeten it. The dose of this mixture must be proportioned to the age and sex of the patient, and to the violence of the disease. A wine-glassful may be given to adults every two, four, or six hours.

By Geoffroy's table it appears, that the foſſil alkali has a greater affinity with the marine, than with the vegetable acid. However, marine ſalt diſſolves readily in the lime juice, throws up a white ſcum to the ſurface, and on applying the ear near the veſſel where the experiment is made, a ſmall hiſſing may be heard, ſimilar to that when acids and alcalies are mixed. It would ſeem probable, that part of the marine ſalt is hereby decompoſed.

That vegetable acids and marine ſalt are antiſeptics, has long been known; but their effects when mixed I apprehend to be but lately diſcovered.

Without farther preface, I ſhall proceed to the particular diſeaſes in which they have been adminiſtered, prepared as above.

*Of the Dyſentery.*

The dyſentery is a very frequent diſorder in this and other Weſt-India iſlands; and ſometimes is epidemic, particularly in the rainy ſeaſons, or when proviſions are ſcarce. Amongſt other cauſes of dyſenteries, I have often known the eating of yams not arrived at maturity, as alſo unripe alligator pears, produce a bloody flux.

Dyſen-

Dyſenteries commonly begin with frequent looſe ſtools for a day or two, attended with gripings: by degrees, the gripes grow more ſevere; nothing is voided by ſtool but a ſmall quantity of mucus, mixed with blood; a teneſmus comes on, and is exceedingly troubleſome.

The appetite fails, the patients are low ſpirited, and ſuffer a great proſtration of ſtrength. The mouth and tongue are much furred and ſlimy, and the taſte is like that of rotten butcher's meat. The deſire of drink is ſometimes exceſſive, but for the moſt part very moderate. The pulſe is very low, feeble, and undulating; and rarely riſes ſo high, as to indicate the uſe of a lancet. Such was the dyſentery in 1771. It proved fatal to many people, both old and young, though treated according to the moſt approved methods of cure; and the loſs of ſeveral patients of mine, convinced me of the neceſſity of uſing antiſeptics early in this diſeaſe.

A vomit ſeemed neceſſary to clear the ſtomach; and ſome gentle purge, to carry off part of the offending matter by ſtool. But the action of theſe, however mild, often increaſed the proſtration of ſtrength, and rendered the ftools fooner bloody. Nor was opium of any real ufe. A tea made of Simarouba, had, in fome, a very falutary effect, whilft, in others, it would by no means remain on the fto-mach.

From a confideration of the antifeptic quality of both the marine falt and the vegetable acid, I was induced to make trial of their effects united in the manner above mentioned. The medicine acted like a charm; and I have found, that from the ufe of it, the frequency of the ftools, the gripes, and tenefmus, have foon worn off; the ftools have gradually become of a natural confiftence and quantity; the fpirits, ftrength, and appetite have returned, and the patient has been reftored to perfect health in a very few days.

When the dyfentery was of long ftanding, ftarch clyfters, with a fmall portion of opium, abated the tenefmus.

This medicine was equally ferviceable in diarrhœas.

### *Diabetes.*

As I had fucceeded fo well in the cure of dyfenteries, I was determined to try its effects in the diabetes; feveral opportunities foon offered: but as thefe cafes were accompanied with with other complaints, especially with fevers of the remitting kind, it will be proper first to speak of

### *The Remittent Fever.*

This, by far the most common fever within the tropics, is the least understood, and consequently for the most part badly treated. Strangers who walk much, or work hard in the heat of the sun, are more subject to it than seasoned Europeans, or natives of the country.

Dr. Cleghorn's description of this fever is accurate and just; his method of cure, simple and easy. Every physician, who would wish to practise with success, should be well acquainted with his valuable performance, as also with what Dr. Lind has said on the subject.

It is, then, sufficient here to observe, that remittent fevers are often attended with diarrhoeas, diabetes, and sometimes with a copious discharge of saliva, as if mercury had been previously given. In such circumstances I have never found the bark of service; a few glasses of the above mixture have fully answered the intention, not only by removing these symptoms, but the fever at the same time.

The

The Peruvian bark afterwards, taken with ſome of the ſame mixture, has effectually ſecured the patient from a return of this dangerous malady.

The mixture rarely acted as an aſtringent in this or any other diſorder: but when this effect took place, the interpoſition of ſome lenient purge was deemed neceſſary.

*Belly-ach.*

The belly-ach with inflammatory ſymptoms has frequently occurred in the courſe of my practice. The inflammatory ſymptoms yielded with difficulty to bleeding, ſmall doſes of emetic tartar, a mercurial pill, repeated doſes of caſtor oil, and diluting drinks, with nitre, fomentations, and clyſters. A copious diſcharge of fœtid excrement for the moſt part gives immediate relief.

I have obſerved in many caſes, after excruciating belly-achs, that the ſtools were liquid, white, ſmall in quantity, and very fœtid. The patients being worn out with pain, grew diſpondent, did not care to ſpeak, fell into cold, clammy ſweats, and were very reſtleſs. They complained of an ill taſte in their mouths. Their tongues were much furred. Their breath was offenſive; and they had a great propenſity to vomit.]

Formerly I attempted the relief of thoſe threatening ſymptoms with the bark, in various forms, as well as claret, and often ſaved my patient; ſometimes, however, I have failed of ſucceſs. When ſuch caſes fall now under my care, I have immediate recourſe to the anti-ſeptic mixture; nor have I been hitherto diſappointed: the ſtools becoming leſs frequent on the uſe of it, and of a better conſiſtence; the cold ſweats alſo diſappear, and the ſpirits ſoon return, together with an appetite for food.

### *The Putrid Sore Throat.*

In June 1770, the putrid ſore throat made conſiderable havock amongſt adults and children. It attacked thoſe of a lax habit, who for a few days had ſlight head-achs, chillineſs and heats alternately, and an uneaſineſs about their throats, but not ſo much as to hinder their ſwallowing.

On examination, the mouth, tongue, and gums were foul and ſlimy; the tonſils and uvula covered with white ſpecks or ſloughs; the breath was hot and offenſive; the ſkin felt hot and pungent to the touch; the pulſe low and quick; quick; a diarrhœa often attended, and the patients were, in general, much dejected.

Antimonial wine with cordials and nourishing diet succeeded best, till the sloughs or spots were removed and separated; then the bark completed the cure. When a diarrhœa accompanied this disorder, I gave the mixture with success.

In all disorders where a gargle is necessary, I make use of the above mixture in preference to any other; and I find it speedily cleanses the tongue, gums, and fauces, and sweetens the breath.

Where lemons or limes cannot be had, vinegar or cream of tartar may be substituted in their room.

From what has been said, it is evident, that the medicine is possessed of considerable antiseptic power; and that its virtue consists in correcting the peccant matter in the stomach and intestinal canal.

All the diseases in which I have given it, had a putrid tendency. I shall be happy to hear of its success in your western hemisphere.

And am, with esteem, Sir,

Your most humble servant,

William Wright.

